# What are We to Understand by the Term Homœpathy

**Published:** 1895-07

**Authors:** J. R. Haynes

**Affiliations:** Indianapolis, Ind.


### WHAT ARE WE TO UNDERSTAND BY THE TERM HOMŒOPATHY?

J. R. Haynes, M. D., Indianapolis, Ind.

When Hahnemann discovered the law of *similia*, he looked about for a name to apply to it that should coincide with the adjustment of that law; hence he adopted the name of Homœopathy, which he took from two Greek words, *omoeon* and *pathos*—*omoeon*, similar, and *pathos*, disease, which he converted into "Homœopathicher."

He makes frequent use of the word in all of his writings to represent the meaning of the law, but nowhere does he use (that I can find) the Latin phrase *similia similibus curanter;* for the word Homœopathischer represents the same meaning, hence it would be using three words to represent one. He does not use the word "ome-on," which many of our friends have made into Home-pathy, which means quite a different thing, but he condemns the phrase in the strongest terms, the attempt to throw out the diphthong, as has been done by those who claim that

Homœopathy is a system and not a law, and by it claim the right to use any remedy or concoction of remedies, and even crude drugs or external applications that may suit their fancy, or the alternate use of remedies, even as many as five or six at a time. They claim that Homœopathy will do in some cases, but in others they have the right to do as they please. Now if one drug is homœopathic to the disease then all of the others are at least superfluous, and do harm to the case and can be of no benefit. This only shows their ignorance, not only of the law, but of its application to the curing of disease.

All remedies have their sphere of action, they cannot go outside of that sphere whether they be a high or a low potency or a crude drug. It must be truly homœopathic to the case in hand or it cannot produce a cure, and much less when more than one drug is used at the same time or in alternation. The extra drug is not only superfluous, but is of positive injury to the other, if it be homœopathic to the case, and destroys the action of the right one.

All of this Hahnemann condemns in the most strenuous manner in all of his writings, teachings, and practice. We all know, who have given proper thought to the subject, that Hahnemann was perfectly right, and that we cannot improve on what Hahnemann has repeatedly told us about following the law; that it must be followed to the letter if we expect to gain the results which he did in making cures. We all know this, and have proved it time and again. Then why should we go "whoring after other gods"? Is it because we are too indolent to take the trouble to learn to apply the law as Hahnemann did and tells us to do if we expect to obtain the results he did? We are perfectly aware that it requires a vast amount of study to make us familiar with the minutiæ of drug action, as well as the cumulative action of disease, and if we expect to find a royal road to the knowledge "on flowery beds of ease," then we have made a mistake, and the sooner we look for some other means of a livelihood the better will it be for those in distress who may fall into our hands seeking relief.

Then does it not stand us in hand to unfurl our banner to the breeze inscribed that we are true to the law, and that upon or under no circumstances will we depart from the ways of truth, let come what may, that it is our light by day and night forever?

---

### The Relative Value of Symptoms.*

SAMUEL A. KIMBALL, M. D., BOSTON, MASS.

* See June number, page 250.

